# Supplementary material for: “Molecular insights into the bactericidal toxin Tle1 of Pseudomonas aeruginosa: Interaction with VgrG, its adaptor, and its immunity protein”
Source: J Biol Chem. 2026 Mar 31;302(5):111416. doi: 10.1016/j.jbc.2026.111416 (PMC13133936; doi:10.1016/j.jbc.2026.111416)
Supplement: Supplementary Material — 2 [file mmc2.docx]

“Molecular Insights into the bactericidal Toxin Tle1 of *Pseudomonas aeruginosa*: Interaction with VgrG, its adaptor, and its immunity protein"

Delphine Lefebvre, Chantal Soscia, Laura Schmitt, Adeline Goulet, Bérengère Ize and Sophie Bleves

**Supporting data:**

**Figure S1:** SignalP 6.0 predictions for (A) PA3291 (Tli1a) and (B) PA3292 (Tli1b).

**Figure S2:** BLAST alignment between PA3291 (Tli1a) and PA3292 (Tli1b).

**Figure S3:** Bacterial two-hybrid assay.

**Figure S4:** Heterologous toxicity test in *E. coli.*

**Figure S5:** Confidence metrics for the AlphaFold 3 models from (A-D) Fig. 3E (a monomer of Tle1 and a monomer of Tli1a) and (E-F) from Fig. 3F (a monomer of Tle1).

**Figure S6**: Confidence metrics for the AlphaFold 3 model from Fig. 5 (trimer of VgrG4a, a monomer of Tle1 and a monomer of Tla1).

**Figure S7:** Each C-terminal extension of VgrG4a recruits a Tle1 and its chaperone Tla1 in a VgrG₃ (residues 511-688) –Tle1₃–Tla1₃ complex.

**Supporting Materials and Methods**

**Table S1**: *E. coli* and *P. aeruginosa* strains, plasmids and oligonucleotides used in Figures 1-8

**Table S2:** *E. coli* and *P. aeruginosa* strains, plasmids and oligonucleotides used exclusively in Figures S3 and S6

**Table S1: *E. coli* and *P. aeruginosa* strains, plasmids and oligonucleotides used in this study**

|  | **Genotype or description** | | **Origin** |  |
| --- | --- | --- | --- | --- |
| **Strains** |  | |  |  |
| ***E. coli*** |  | |  |  |
| DH5⍺ | F-, Δ(*argF* -*lac* )U169, *phoA* , *supE44* , Δ(*lacZ*)M15, *relA* , *endA* , *thi* , *hsdR* | | Invitrogen |  |
| CC118λpir | ∆(*ara*-*leu* ) *ara* D ∆ *lac* X74 *gal* E *gal* K *pho* A20 *thi* -1 *rps* E *rpo* B *arg*E (Am) *rec* A1 RfR (λpir) | | Laboratory collection |  |
| BL21(DE3)pLysS | F– *ompT* *gal* *dcm* *lon* *hsdSB*(*rB*–*mB*– ) λ(DE3 [*lacI* *lacUV5*-T7p07 *ind1* *sam7* *nin5* ]) [*malB*+]K-1 2(λS) *pLysS*[*T7p20* *orip15A*, CmR | | Laboratory collection |  |
| BTH101 | F-, *cya-99, araD139, galE15, galK16, rpsL1 (Str r), hsdR2, mcrA1, mcrB1* | | Laboratory collection |  |
| ***P. aeruginosa*** |  | |  |  |
| PAO1 | Wild-type prototroph | | Laboratory collection |  |
| PAO1 *tli1a* V5 | chromosomally encoded *tli1aV5* translational fusion in PAO1 | | This work |  |
| PAO1 *tli1b* V5 | chromosomally encoded *tli1bV5* translational fusion in PAO1 | | This work |  |
| **Plasmids** |  | |  |  |
| **pETDuet-1** | Expression vector, *ColE1* origin, *lacI*, *PT7* , ApR | | Novagen |  |
| **pET22b(+)** | Expression vector, PBR322 origin, PT7, PelB signal sequence, ApR | | Novagen |  |
| pSBC103 | *tle1* cloned into pETDuet-1, C-terminal 6His epitope, ApR | | This work |  |
| pSBC104 | *tle1* cloned downstream the PelB signal sequence in pET22b(+), C-terminal 6His epitope, ApR | | This work |  |
| pDL45 | *tla1* cloned into pETDuet-1, C-terminal 6his epitope, ApR | | This work |  |
| **pRSFDuet-1** | Expression vector, *RSF* origin, *lacI*, *PT7*, KanR | | Novagen |  |
| pSBC105 | *tli1a* cloned into pRSFDuet-1, C-terminal V5 epitope, KanR | | This work |  |
| pDL44 | *tla1* cloned into pRSFDuet-1, C-terminal Strep epitope, KanR | | This work |  |
| pDL46 | *vgrG4a* (sequence coding residues 511-688) cloned into pRSFDuet-1, C-terminal Strep epitope, KanR | | This work |  |
| pDL47 | *tli1a* (sequence coding residues 31-184) signal sequence less cloned into pRSFDuet-1, C-terminal V5 epitope, KanR | | This work |  |
| **pUT18** | Bacterial Two Hybrid vector, *ColE1* origin, *Plac*, T18 fragment of *Bordetella* *pertussis* *CyaA*, AmpR | | Euromedex |  |
| pDL12 | *tle1 (PA3290)* cloned upstream the T18 coding sequence in pUT18 | | This work |  |
| pDL18 | *tla1 (PA3293*) cloned upstream the T18 coding sequence in pUT18 | | This work |  |
| pDL20 | *vgrG4a* (sequence coding residues 511-688) (*PA3294*) cloned upstream the T18 coding sequence in pUT18 | | This work |  |
| pDL22 | *tli1a (PA3291*) (sequence coding residues 31-184) cloned upstream the T18 coding sequence in pUT18 | | This work |  |
| pDL24 | *tli1b (PA3292)* (sequence coding residues 21-285) cloned upstream the T18 coding sequence in pUT18 | | This work |  |
| pDL39 | *tle1 (PA3290)* (sequence coding residues 1-450) cloned upstream the T18 coding sequence in pUT18 | | This work |  |
| pDL43 | *tle1 (PA3290)* (sequence coding residues 451-882) cloned upstream the T18 coding sequence in pUT18 | | This work |  |
| T18-Pal | *pal* cloned upstream the T18 coding sequence in pUT18 | | Battesti *et al*., 2012 (45) |  |
| **pKNT25** | Bacterial Two Hybrid vector, *p15A* origin, *Plac* , T25 fragment of *Bordetella pertussis cyaA*, KanR | | Euromedex |  |
| pDL11 | *tle1 (PA3290)* cloned upstream the T25 coding sequence in pKNT25 | | This work |  |
| pDL17 | *tla1 (PA3293)* cloned upstream the T25 coding sequence in pKNT25 | | This work |  |
| pDL21 | *vgrG4a* (sequence coding residues 511-688) (*PA3294*) cloned upstream the T25 coding sequence in pkNT25 | | This work |  |
| pDL23 | *tli1a* (*PA3291*) (sequence coding residues 31-184) cloned upstream the T25 coding sequence in pKNT25 | | This work |  |
| pDL25 | *tli1b (PA3292)* (sequence coding residues 21-285) cloned upstream the T25 coding sequence in pKNT25 | | This work |  |
| pDL51 | *tla1*_R197A_ cloned upstream the T25 coding sequence in pKNT25 | | This work |  |
| pDL54 | *tla1*_R197A, R92A_ cloned upstream the T25 coding sequence in pKNT25 | | This work |  |
| pDL58 | *tla1*_R197A, R92A, D242A_ cloned upstream the T25 coding sequence in pKNT25 | | This work |  |
| pDL55 | *vgrG4a_E673A, E687A_* (sequence coding residues 511-688) cloned upstream the T25 coding sequence in pKNT25 | | This work |  |
| pDL59 | *vgrG4a_K679A, R688A, E687A_ (*sequence coding residues 511-688) cloned upstream the T25 coding sequence in pKNT25 | | This work |  |
| **pUT18C** | Bacterial Two Hybrid vector, *ColE1* origin, *Plac*, T18 fragment of *Bordetella* *pertussis* *CyaA*, AmpR | | Euromedex |  |
| pSD3 | *tle1 (PA3290)* cloned downstream the T18 coding sequence in pUT18C | | This work |  |
| pSD5 | *tli1a (PA3291)* (sequence coding residues 31-184) cloned downstream the T18 coding sequence in pUT18C | | This work |  |
| pSD7 | *tli1b* (*PA3292*) (sequence coding residues 21-285) cloned downstream the T18 coding sequence in pUT18C | | This work |  |
| pSD9 | *tla1* (*PA3293*) cloned downstream the T18 coding sequence in pUT18C | | This work |  |
| pDL8 | *vgrG4a* (sequence coding residues 511-688) *(PA3294)* cloned downstream the T18 coding sequence in pUT18C | | This work |  |
| pDL61 | *tle1*_R37A_ *(PA3290)* cloned downstream the T18 coding sequence in pUT18C | | This work |  |
| **pKT25** | Bacterial Two Hybrid vector, *p15A* origin, *Plac* , T25 fragment of *Bordetella* *pertussis* *CyaA*, KanR | | Euromedex |  |
| pSD2 | *tle1 (PA3290*) cloned downstream the T25 coding sequence in pKT25 | | This work |  |
| pSD4 | *tli1a (PA3291)* (sequence coding residues 31-184) cloned downstream the T25 coding sequence in pKT25 | | This work |  |
| pSD6 | *tli1b (PA3292)* (sequence coding residues 21-285) cloned downstream the T25 coding sequence in pKT25 | | This work |  |
| pSD8 | *tla1 (PA3293)* cloned downstream the T25 coding sequence in pKT25 | | This work |  |
| pDL7 | *vgrG4a* (sequence coding residues 511-688) (*PA3294*) cloned downstream the T25 coding sequence in pKT25 | | This work |  |
| TolB-T25 | *tolB* cloned downstream the T25 coding sequence in *pkT25* | | Battesti *et al.,* 2012 (45) |  |
| **pKNG101** | R6K origin, TRK2 origin, *mobRK2*, *sacBR*+, SmR | | Kaniga *et al.*, 1991 |  |
| pDL50 | suicide vector for *tli1aV5* insertion, SmR | | This work |  |
| pDL31 | suicide vector for *tli1bV5* insertion, SmR | | This work |  |
| pRK2013 | *ColE1 origin, Tra+, mob+, KmR* | | Laboratory collection |  |
| **Plasmids** | **Oligonucleotides names** | **Oligonucleotides sequences** | | |
| pSBC103 | CSO13 | agaaggagatataccatggatgcctaattttggattt | | |
|  | CSO14 | ttatgcggccgcaagcttgtcgactcaatggtgatggtggtgatgaagcgattccatcaggtcct | | |
| pSBC104 | CSO15 | ccggcgatggccatggggcctaattttggatttcat | | |
|  | CSO16 | ggtggtggtgctcgagaagcgattccatcaggtcct | | |
| pDL45 | DLO48 | tcaatggtgatggtggtgatgtgcgcgactcgttcccgccg | | |
|  | DLO48bis | ttatgcggccgcaagcttgtcgactcaatggtgatggtggtgatg | | |
| pDL46 | DLO46 | aggagatataccatgggcgactgggacgagaacatc | | |
|  | DLO47 | ctgcgggtggctccaagcgctgcgctcggcgagcg | | |
|  | DLO47bis strep | ggccgcaagcttgtcgactcatttttcgaactgcgggtggc | | |
| pDL47 | DLO49 | aggagatataccatgggctgccaagctgggcctgaa | | |
|  | DLO50 | gaccgaggagagggttagggataggcttaccgttagccggacatatcgt | | |
|  | DLO14bis V5 | ggccgcaagcttgtcgactcacgtagaatcgagaccgaggagagggt | | |
| pDL11, pDL12 | DLO5 | ccaagcttgcatgcctgcagatgcctaattttggat | | |
|  | DLO6 | cggtacccggggatcctcaagcgattccatcag | | |
| pDL17, pDL18 | DLO21up | ccaagcttgcatgcctgcaggacgccgaacccccgccat | | |
|  | DLO22dw | ggtacccggggatcctctagagttgcgcgactcgttcccgc | | |
| pDL20, pDL21 | DLO31 | ccaagcttgcatgcctgcagggactgggacgagaacatc | | |
|  | DLO32 | ggtacccggggatcctctagagtgcgctcggcgagcgcctg | | |
| pDL22, pDL23 | DLO29 | ccaagcttgcatgcctgcaggtgccaagctgggcctgaa | | |
|  | DLO30 | ggtacccggggatcctctagagtgttagccggacatatcgt | | |
| pDL24, pDL25 | DLO27 | ccaagcttgcatgcctgcaggtgccagagcgggccggacat | | |
|  | DLO28 | ggtacccggggatcctctagagttacgttttctccagccatct | | |
|  |  |  | | |
| pDL43 | DLO43 | ccaagcttgcatgcctgcaggctcgccacccgtttcaac | | |
|  | DLO41 | ggtacccggggatcctctagagtaagcgattccatcaggtc | | |
| pDL51 | DLO68 | ggtcgctgccttggccgcctggagcg | | |
|  | DLO69 | ctcgctccaggcggccaaggcagcgacc | | |
| pDL54 | DLO70 | gcatcgactgcgcccgccacctgatg | | |
|  | DLO71 | catcaggtggcgggcgcagtcgatgc | | |
| pDL58 | DLO80 | cagggcttgcaggccttcgaccagcgc | | |
|  | DLO81 | gcgctggtcgaaggcctgcaagccctg | | |
| pDL55 | DLO72 | ccggtctgcgaagcgtgcctgctacag | | |
|  | DLO73 | ctgtagcaggcacgcttcgcagaccgg | | |
|  | DLO74 | ccaggcgctcgccgcgcgcactctagagg | | |
|  | DLO75 | ctagagtgcgcgcggcgagcgcc | | |
| pDL59 | DLO76 | cctgctacaggcggcgaaaagaggccaggc | | |
|  | DLO77 | gcctggcctcttttcgccgcctgtagcaggca | | |
|  | DLO78 | ggcgctcgccgaggccactctagaggat | | |
|  | DLO79 | atcctctagagtggcctcggcgagcgcc | | |
|  | DLO82 | ggcgctcgccgcggccactctag | | |
|  | DLO83 | ctagagtggccgcggcgagcgcc | | |
| pSD3 | SDO19 | cgcagtggaacgccactgcaggcctaattttggatttcat | | |
|  | SDO20 | cggtacccggggatcctctagatcaaagcgattccatcag | | |
| pSD5 | SDO23 | cgcagtggaacgccactgcaggtgccaagctgggcctgaa | | |
|  | SDO24 | cggtacccggggatcctctagatcagttagccggacatat | | |
| pSD7 | SDO27 | cgcagtggaacgccactgcaggtgccagagcgggccggac | | |
|  | SDO28 | cggtacccggggatcctctagatcatacgttttctccagc | | |
| pSD9 | SDO31 | cgcagtggaacgccactgcaggacgccgaacccccgccat | | |
|  | SDO32 | cggtacccggggatcctctagatcatgcgcgactcgttcc | | |
| pDL8 | SDO35-2 / UT18C-G4DUF2345 | cgcagtggaacgccactgcagggactgggacgagaacatc | | |
|  | SDO36 | cggtacccggggatcctctagatcagcgctcggcgagcgc | | |
| pDL61 | DLO84 | tattaaagcagcagagcgatgaagcggttttttcaagggcg | | |
|  | DLO85 | cgcccttgaaaaaaccgcttcatcgctctgctgctttaata | | |
| pSD2 | SDO17 | gcgcacgcggcgggctgcagggcctaattttggatttcat | | |
|  | SDO18 | aggtacccggggatcctctagatcaaagcgattccatcag | | |
| pSD4 | SDO21 | gcgcacgcggcgggctgcagggtgccaagctgggcctgaa | | |
|  | SDO22 | aggtacccggggatcctctagatcagttagccggacatat | | |
| pSD6 | SDO25 | gcgcacgcggcgggctgcagggtgccagagcgggccggac | | |
|  | SDO26 | aggtacccggggatcctctagatcatacgttttctccagc | | |
| pSD8 | SDO29 | gcgcacgcggcgggctgcagggacgccgaacccccgccat | | |
|  | SDO30 | aggtacccggggatcctctagatcatgcgcgactcgttcc | | |
| pDL7 | SDO33-2 / KT25-G4DUF2345 | gcgcacgcggcgggctgcaggggactgggacgagaacatc | | |
|  | SDO34 | aggtacccggggatcctctagatcagcgctcggcgagcgc | | |
| pDL50 | DLO58 | caggtcgacggatccccgggtatgcgccgtttcatcctga | | |
|  | DLO59 | atcgagaccgaggagagggttagggataggcttaccgttagccggaca | | |
|  | DLO60 | tatccctaaccctctcctcggtctcgattctacgtgaaggcaggtgggaa | | |
|  | DLO61 | tatgcatccgcgggcccgggaaccaccagtgagcgtactg | | |
| pDL31 | DLO35 | caggtcgacggatccccgggataaagatgattacttgaga | | |
|  | DLO36 | atcgagaccgaggagagggttagggataggcttacctacgttttctcc | | |
|  | DLO37 | cctatccctaaccctctcctcggtctcgattctacgtgagagagcatgggct | | |
|  | DLO38 | tatgcatccgcgggcccgggaaccgcccatatttatctc | | |

**Supporting Material and Methods**

***In silico* signal peptide prediction**

SignalP 6.0 (<https://services.healthtech.dtu.dk/services/SignalP-6.0/>) was used to identify putative lipoprotein signal peptide sequences in the corresponding protein sequences. The slow mode was selected to achieve greater accuracy.

**Protein–protein interaction *in silico* analyses**

AlphaFold 3 predictions were generated with predicted aligned errors (PAE) and predicted local distance difference test (pLDDT) values as confidence metrics. All five models produced were used to assess the reliability of the predictions. AlphaBridge was then applied to the AlphaFold-predicted protein complex models to analyze protein–protein interfaces and evaluate their confidence using the predicted interaction Confidence Score (piCS) with a default cutoff of 0.7. AlphaBridge generated a network displaying each interface along with its corresponding piCS value.

**Table S2:** *E. coli* and *P. aeruginosa* strains, plasmids and oligonucleotides used exclusively in Figures S3 and S6

|  | **Genotype or description** | | **Origin** |
| --- | --- | --- | --- |
| **Plasmids** |  | |  |
| **pRSFDuet-1** | Expression vector, *RSF* origin, *lacI*, *PT7*, KanR | | Novagen |
| pSBC105 | *tli1a* cloned into pRSFDuet-1, C-terminal V5 epitope, KanR | | This work |
| pSBC113 | *tli1b* cloned into MCS1 (C-terminal Strep epitope) and *tli1a* cloned into MCS2 (C-terminal V5 epitope) in pRSFDuet-1, KanR | | This work |
| pDL4 | *tli1b* cloned into pRSFDuet-1, C-terminal Strep epitope, KanR | | This work |
| **pUT18** | Bacterial Two Hybrid vector, *ColE1* origin, *Plac*, T18 fragment of *Bordetella* *pertussis* *CyaA*, AmpR | | Euromedex |
| pDL39 | *tle1 (PA3290)* (sequence coding residues 1-450) cloned upstream the T18 coding sequence in pUT18 | | This work |
| pDL43 | *tle1 (PA3290)* (sequence coding residues 451-882) cloned upstream the T18 coding sequence in pUT18 | | This work |
| **pUT18C** | Bacterial Two Hybrid vector, *ColE1* origin, *Plac* , T18 fragment of *Bordetella* *pertussis* *CyaA*, AmpR | | Euromedex |
| pDL37 | *tle1 (PA3290)* (sequence coding residues 1-450) cloned downstream the T18 coding sequence in pUT18C | | This work |
| pDL41 | *tle1 (PA3290)* (sequence coding residues 451-882) cloned downstream the T18 coding sequence in pUT18C | | This work |
| **Plasmids** | **Oligonucleotides**  **names** | **Oligonucleotides sequences** | |
| pSBC105 | CSO11 | aaggagatatacatatgcagaagttgatgagttt | |
|  | CSO12 | gcgtggccggccgatatctcacgtagaatcgagaccgaggagagggttagggataggcttaccgttagccggacatatcgt | |
| pSBC113 | CSO60 | gcgcctgcaggtcgactgaaaggagatatacatgaacgggcgcggc | |
|  | CSO61 | ccgcaagcttgtcgactcatttttcgaactgcgggtggctccaagcgcttacgttttctccagc | |
|  | CSO31 | ctcgattctacgtgagatatctttaagaaggagatatacatat | |
|  | CSO32 | gcgtggccggccgatatctcagtggtggtggtggtggtg | |
|  | CSO11 | aaggagatatacatatgcagaagttgatgagttt | |
|  | CSO12 | gcgtggccggccgatatctcacgtagaatcgagaccgaggagagggttagggataggcttaccgttagccgga | |
| pDL4 | DLO1 | aggagatataccatggatgaacgggcgcggcatcggcct | |
|  | DLO2 | tcatttttcgaactgcgggtggctccaagcgcttacgttttctccagc | |
|  | DLO3 | ccgcaagcttgtcgactcatttttcgaactgcgggtggctccaagcgct | |
| pDL37 | SDO19 | cgcagtggaacgccactgcaggcctaattttggatttcat | |
|  | DLO39 | gtacccggggatcctctagagtttcttcggagacgctgaa | |
| pDL39 | DLO5 | ccaagcttgcatgcctgcagatgcctaattttggat | |
|  | DLO39 | gtacccggggatcctctagagtttcttcggagacgctgaa | |
| pDL41 | DLO42 | cgcagtggaacgccactgcagggctcgccacccgtttcaac | |
|  | DLO41 | ggtacccggggatcctctagagtaagcgattccatcaggtc | |
| pDL43 | DLO43 | ccaagcttgcatgcctgcaggctcgccacccgtttcaac | |
|  | DLO41 | ggtacccggggatcctctagagtaagcgattccatcaggtc | |

**Supplementary Reference:**

Kaniga, K., Delor, I., and Cornelis, G.R. (1991) A wide-host-range suicide vector for improving reverse genetics in gram-negative bacteria: inactivation of the *blaA* gene of *Yersinia enterocolitica*, *Gene*. (1991) 109(1):137-41.
